# Concomitant Cryptococcosis and Burkholderia Infection in an Asymptomatic Lung Transplant Patient with Cystic Fibrosis

**Published:** 2010-11-01

**Authors:** S. Shafaghi, M. Pour Abdollah, P. Tabarsi, F. Ghorbani, S. S. M. Makki, H. R. Khoddami Vishteh, J. Faeghi, K. Najafizadeh

**Affiliations:** 1 *General Practitioner, Organ Donation Research Center of Iran; *; 2 *Lung Transplantation Research Center; *; 3 *Associated Professor of Pulmonology and Critical Care Medicine; Lung Transplantation Research Center, National Research Institute of Tuberculosis and Lung Disease, Masih Daneshvari Hospital, Shahid Beheshti University of Medical Sciences, Tehran, Iran*

**Keywords:** Cryptococcosis, Burkholderia infection, Cystic fibrosis, Double lung transplantation

## Abstract

Concomitant pulmonary infections with *Cryptococcus neoformans* and *Burkholderia cepacia* in lung transplant recipients are very rare and create unique diagnostic and therapeutic dilemmas. Herein, we present a double lung transplant patient with cystic fibrosis who was found to have coinfection with these two rare organisms, though he was completely asymptomatic.

## INTRODUCTION

Pulmonary cryptococcosis is caused by inhalation of *Cryptococcus neoformans*. It is well-known that pulmonary cryptococcal infection tends to occur in immunocompromised individuals, although it can sometimes happen even in immunocompetent hosts [1-4].

Although cryptococcosis occurs rarely after lung transplantation, it is the third most commonly occurring invasive fungal infection (after candidiasis and aspergillosis) in solid organ transplantation (SOT) recipients [5]. Early diagnosis and treatment of cryptococcosis are very important to improve survival. However, early signs of infection may be masked due to diminished inflammatory response.

Approximately, 2.8%–5% of SOT recipients develop infection with *C. neoformance*, with overall mortality ranging from 20% to 42%; the mortality in patients with central nervous system infection approaches 50% [5].

*Burkholderia cepacia* is another important pathogen causing pulmonary infections in people with cystic fibrosis (CF). The organism possesses antibiotic resistance and associates with high mortality [6].

Herein, we introduce a double lung transplant patient with cystic fibrosis who was found to have two rare infections concomitantly, though he was completely asymptomatic.

## CASE REPORT

A 21-year-old man with cystic fibrosis and end-stage bronchiectasis was transplanted in 2008. No induction therapy was given. The patient was given prednisolone, cellcept, and cyclosporine. CMV prophylaxis with gancyclovir for 14 days and valcyte for 2.5 months after transplantation were prescribed. For fungal prophylaxis, itraconazole was given for three months after surgery. There were no significant complications after transplantation. He had a short contact with his aunt who had been diagnosed to have miliary tuberculosis after kidney transplantation.

**Figure 1:**
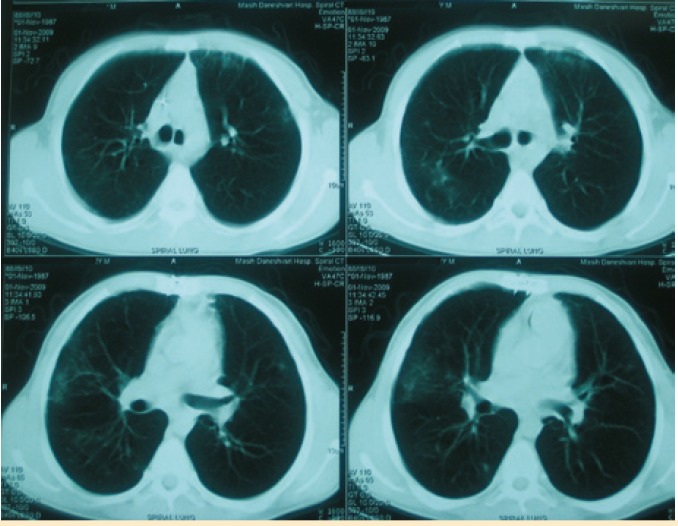
Lung spiral CT on November 1, 2009, showing incidental finding of an infiltration

**Figure 2:**
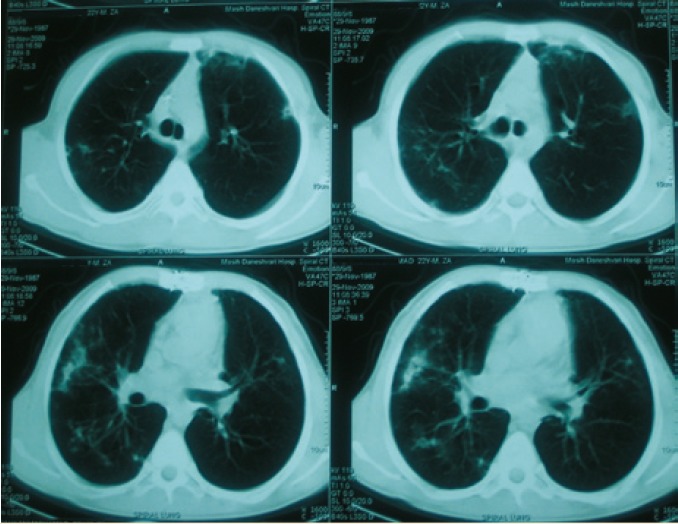
Lung spiral CT on November 29, 2009, showing progression of the infiltration

**Figure 3:**
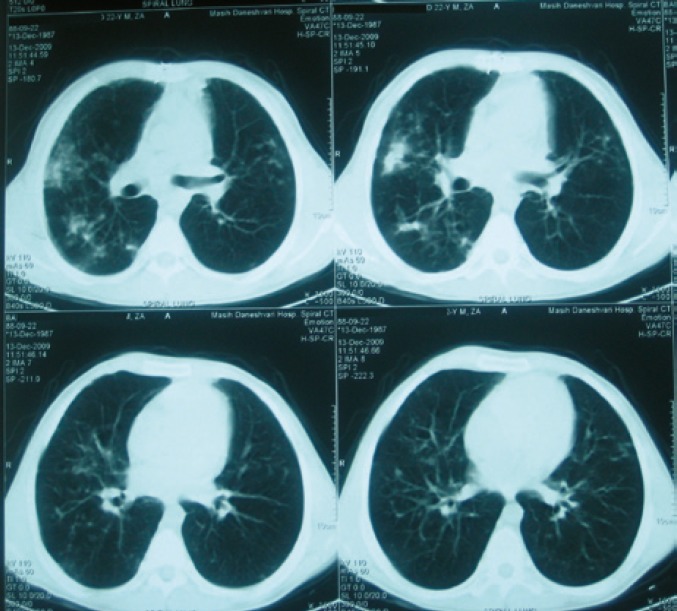
Lung spiral CT on December 13, 2009, showing progression of the infiltration after starting treatment for burkholderia

Routine chest computed tomography (CT) was performed for check up one year after transplantation. The patient was completely asymptomatic. There was infiltration and nodularity in the superior segment of the lower lobe of right lung (Fig 1). Bronchoscopy revealed no purulent secretion in bronchi; all of the cultures were negative for bacteria, fungi, tuberculosis, and viruses. Trans-bronchial lung biopsy was normal. Patient was then observed closely. As the patient had had contact with a patient with tuberculosis, a purified protein derivative (PPD) test was done which was found negative. One month later, another CT was performed. The infiltration was progressed and there were also some infiltrations in the right and left upper lobes (Fig 2). Another bronchoscopy found purulent secretions in the superior segment of the right lower lobe (RLL). Bronchoalveolar lavage was positive for *B. cepacia*. The subtype could not be determined because of limited facilities. In antibiogram, the organism was found sensitive only to ciprofloxacin. Although co-trimoxazole is one of the best modalities for controlling *Burkholderia*, we did not prescribe it because of the possibility of resistance to co-trimoxazole due its continuous use for PCP prophylaxis. Therefore, a combination therapy with meropenem (1 g tid) plus ciprofloxacin (400 mg tid) for 14 days and doxycycline and inhaled tubramycin for three months were administered. Chest CT after two weeks did not show any changes (Fig 3) but in bronchoscopy, there was no more purulent secretions which reflected response to antibiotic therapy. Bronchoscopy was repeated; there was no bacterial or fungal growth in bronchoalveolar lavage culture. TBLB, at that time, showed patchy interstitial infiltration and grade A2 acute rejection, but because the patient was still completely asymptomatic and since oxygenation and pulmonary function were perfect, no treatment was given.

As patient was suffering from CF, paranasal sinuses were also evaluated. CT showed complete opacification of all paranasal sinuses similar to that in pre-transplantation except that the patient did not have any posterior nasal discharge nor any other symptoms; then, only antibiotic therapy for the lung problem looked enough.

**Figure 4:**
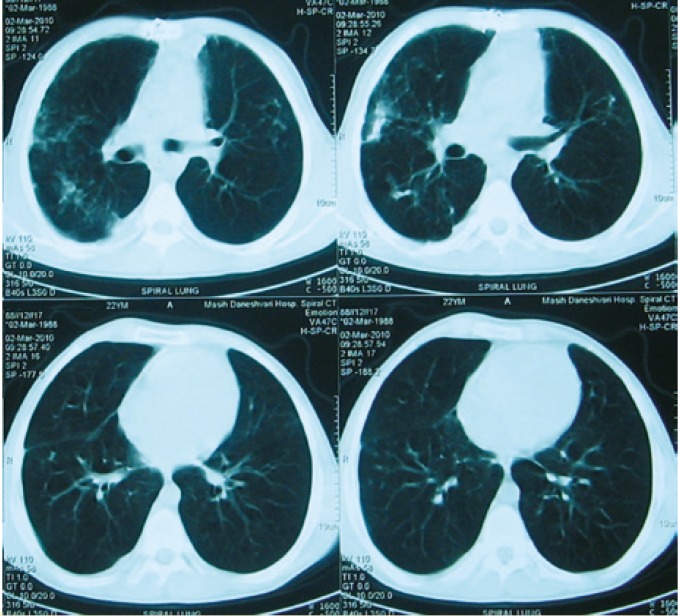
Lung spiral CT on March 8, 2010 showing partial improvement of the infiltration after starting treatment for cryptococcosis

One month after start of treatment for *Burkholderia*, CT showed progression of the infiltration. CT-guided biopsy was performed by west cot 20G needles. Sample smear and cultures were negative for *Burkholderia* and also for other bacteria and fungi. Pathologically, it was inadequate. Then open lung biopsy was considered. A wedge biopsy was taken from the superior segment of RLL. It showed some necrosis associated with histiocytic infiltration and presence of some poorly preserved yeast-like structures suspicious for cryptococcal infection. There was no important pathology in pleural biopsy. Latex agglutinin test revealed cryptococcal antigen in both cerebrospinal fluid (CSF) and serum. Liposomal amphotericin and fluconasol were administered and after two weeks, only fluconasol was continued for one year. While the patient is still completely asymptomatic and his lung function is normal, the treatment for *Burkhorderia* and *Cryptococcus* is continuing with inhaled tubramycin and fluconasol, respectively. CT, three months after the initiation of cryptococal treatment shows almost 30% improvement (Fig 4); six months after the initiation of cryptococal treatment, it shows about 40% improvement. 

## DISCUSSION

We introduced a lung transplant patient who was completely asymptomatic in spite of having both burkholderia and cryptococcal infections together.

Considering the fact that burkholderia and cryptococcal infections are very rare, there is no report on coinfection with these two organisms in lung transplant patients.

*B. cepacia* is an important pathogen causing pulmonary infection in people with CF due to its antibiotic resistance and its high mortality.

During the first three months post-transplantation, *B. cepacia* disease generally manifest as progressive infiltrates on chest radiographs, with increasing alveolar-arterial oxygen gradients, sepsis, and death despite multiple intravenous antibiotics. *B. cepacia* infection has been associated with bronchiolitis oblitrance, abscesses formation, pneumonia and acute renal failure between 14 and 48 months post-transplantation [7,8]. We could not find any report of asymptomatic *B. cepacia* infection in the literature.

Pulmonary cryptococcosis may be detected as an incidental finding in asymptomatic patients [9,10]. Approximately 53%–72% of the SOT recipients with cryptococcosis have disseminated disease or central nervous system (CNS) involvement [11,12]. Overall, 61% of the SOT recipients in one report had disseminated disease; 54% had pulmonary and 8.1% had skin, soft tissue or osteoarticular cryptococcosis [10]. In one report, patients with CNS disease were more likely to be fungemic than those without CNS disease [13].

Our study showed that taking history and physical examination of immunocopromised patients are not reliable enough and it is necessary to perform surveillance CT for all of the lung transplant patients at least one year after transplantation and considering aggressive diagnostic procedures such as open lung biopsy for definite diagnosis and treatment of opportunistic infections.

Concomitant pulmonary infections with *C. neoformans* and *B. cepacia* in lung transplant recipients are rare and create unique diagnostic and therapeutic dilemmas. Prolonged antibiotic therapy before transplantation in those with CF may cause infection with uncommon antibiotic-resistant organisms. Newer diagnostic methods allow for detecting these emerging organisms. Considering these opportunistic infections in the differential diagnosis of transplant recipients infections and performing complete identification and susceptibility tests are critical for providing appropriate treatment.
